# Measuring the inequalities in healthcare resource in facility and workforce: A longitudinal study in China

**DOI:** 10.3389/fpubh.2023.1074417

**Published:** 2023-03-16

**Authors:** Enhong Dong, Xiaoting Sun, Ting Xu, Shixiang Zhang, Tao Wang, Lufa Zhang, Weimin Gao

**Affiliations:** ^1^Department of Health Management, School of Nursing and Health Management, Shanghai University of Medicine and Health Science, Shanghai, China; ^2^Health and Medical Communication Research Center, School of Media and Communication, Shanghai Jiao Tong University, Shanghai, China; ^3^Institute of Healthy Yangtze River Delta, Shanghai Jiao Tong University, Shanghai, China; ^4^College of Public Health and Family Medicine, Shanghai Tongji Hospital, Tongji University School of Medicine, Shanghai, China; ^5^Emergency Medical Rescue Technology Research Institute, Shanghai University of Medicine and Health Science, Shanghai, China; ^6^Department of Emergency Medicine, Shanghai Tongji Hospital, Tongji University School of Medicine, Shanghai, China; ^7^Department of Public Economy and Social Policy, School of International and Public Affairs, Shanghai Jiao Tong University, Shanghai, China; ^8^Department of Pharmacy, School of Pharmaceutical Sciences and Yunnan Key Laboratory of Pharmacology for Natural Products, Kunming Medical University, Kunming, China

**Keywords:** healthcare resource, distribution, inequality, Theil index, spatial autocorrelation analysis

## Abstract

**Objective:**

The study aimed to measure time trends of inequalities of the geographical distribution of health facilities and workforce in Shanghai from 2010 to 2016 and used a spatial autocorrelation analysis method to precisely detect the priority areas for optimizing health resource reallocation in metropolises like Shanghai in developing countries.

**Methods:**

The study used secondary data from the Shanghai Health Statistical Yearbook and the Shanghai Statistical Yearbook from 2011 to 2017. Five indicators on health resources, namely, health institutions, beds, technicians, doctors, and nurses, were employed to quantitatively measure the healthcare resource in Shanghai. The Theil index and the Gini coefficient were applied to assess the global inequalities in the geographic distribution of these resources in Shanghai. Global and local spatial autocorrelation was performed using global Moran's index and local Moran's index to illustrate the spatial changing patterns and identify the priority areas for two types of healthcare resource allocation.

**Results:**

Shanghai's healthcare resources showed decreasing trends of inequalities at large from 2010 to 2016. However, there still existed an unchanged over-concentration distribution in healthcare facilities and workforce density among districts in Shanghai, especially for doctors at the municipal level and facility allocation at the rural level. Through spatial autocorrelation analysis, it was found that there exhibited a significant spatial autocorrelation in the density distribution of all resources, and some identified priority areas were detected for resource re-allocation policy planning.

**Conclusion:**

The study identified the existence of inequality in some healthcare resource allocations in Shanghai from 2010 to 2016. Hence, more detailed area-specific healthcare resource planning and distribution policies are required to balance the health workforce distribution at the municipal level and institution distribution at the rural level, and particular geographical areas (low–low and low–high cluster areas) should be focused on and fully considered across all the policies and regional cooperation to ensure health equality for municipal cities like Shanghai in developing countries.

## Introduction

Health inequality refers to the unjust and avoidable difference in people's health across the whole population and between specific population groups (1, 2). It could induce health equity, which is of great importance to the health system and access to healthcare and can provide the opportunity for enjoying equitable healthcare services among different groups irrespective of their gender, race, economic or political belief, and geographic location. Many scholars have unanimously agreed that access to equitable, effective, and economic healthcare services is important for health promotion, disease prevention, reduction in unnecessary disability, and premature death, and thus securing health equity (3, 4). Equitable geographical distribution of healthcare resources is essential to access healthcare services as it can ensure access for those vulnerable groups who lack affordable transportation (5, 6). Conversely, inequality in healthcare goes against the principles of social justice. It is shaped by social determinants beyond the control of individuals and limits people's opportunities to live longer and healthier lives (7, 8). It means that everyone does not have an equal right to access the highest attainable standard of physical and mental health (9). Previous studies have proved that the uneven distribution of healthcare resources results in growing disparities in wellbeing and health outcomes between the rich and poor (e.g., average life expectancy, morbidity, and mortality) (7–11).

The terms “inequity” and “inequality” can sometimes be confused in their significations as they both point out existing disparities that are unjust and socially unacceptable as well (12, 13), but they cannot be considered synonyms as they suggest different causes underlying similar consequences. Inequity is the unequal consequences of mal-governance, wrongdoings of institutions, or cultural exclusion, whereas inequality indicates that the lack of innate endowment is the key determinant of uneven distribution of resources. Health inequity, or health disparity, is a specific type of health inequality signifying the unfair discrepancies in health, which is systematic and avoidable by plausible approaches. Health inequality, in this sense, is viewed as the quantitative objective description of unfair and legitimate differences that cannot be attributed to any individual responsibility (14). According to Braveman's definition, inequality is referred as “differences in the distribution of resources or outcomes among people due to conditions that can be minimized or modified by policies” (15). Accordingly, health inequality can be reduced by amendable health policies of the governments. Being connected in their meanings, equity, and inequity tend to trigger discussions on social and distributive justice. Inequity raises concerns about unfair and uneven distributions of resources. The application of social and distributive justice principles enables societies to allocate resources based on needs, which often leads to a proportional allocation of resources. In some cases, those allocations might be unfair in terms of equality.

Since the mid-1980s, China has experienced rapid urbanization. Along with the uneven economic development between the east and west of China, and between rural and urban areas, the gaps in healthcare resource distribution enlarged. Compared to the more developed eastern provinces, western and central China have fewer health institutions, facilities, and health staffing (11, 16). Notably, such gaps also exist in areas of different urbanization levels in developed cities. For example in Shanghai, central districts have higher densities of healthcare resources than suburban districts do, reflected in the number of doctors and equipment per 1,000 of the population from 2010 to 2016 (8).

To ensure social justice and address inequalities in healthcare resources, the Chinese government has launched a novel phase of health reform since 2009, aiming to reverse the market-oriented health system into one that ensures easy access to safe, efficient, convenient, equitable, and affordable healthcare services to every household. In compliance with the central reform guidelines, the Shanghai municipal government also made targeted efforts to alleviate disparities in the allocation of healthcare resources between rural and urban areas.

A considerable body of studies has reported the variations in the regional distribution of healthcare resources and inequality of their allocation as a result across China's 31 regions (1, 8, 11, 17–24). Most of them focused on describing the regional distribution gaps (e.g., rural and urban areas) or variations across different levels of medical institutions (e.g., tertiary hospitals and primary health centers) in the quantity and inequality in healthcare resource allocation in China. To the best of our knowledge, little research has been done on examining the change of the differences in geographic distribution and on measuring the inequality of healthcare resource allocation over time since China's 2009 reform (8, 25, 26). Moreover, the identification of priority areas for further re-planning and re-allocation of healthcare resources based on a comprehensive comparison of different types of healthcare resources was also scarce up to now. Furthermore, even though the empirical investigations of inequality in the health workforce or facility distribution have been conducted widely between groups, such as counties, provinces, states, or rural–urban areas, this between-group disparity ratio merely reflected the average density differences between the two strata. They completely ignored the inter-unit (inter-county) density differences within the strata, which could turn out to be quite large.

In doing so, on the basis of our previous research (8, 25), using the same dataset targeting a mega-city in China, this study first examines the trends and differences in the geographical distribution of medical institutions, beds, technicians, doctors, and nurses in Shanghai from 2010 to 2016. Second, this study further measures the inequality of these healthcare resources using the Theil index and spatial statistics for detecting the priority areas to provide evidence for the government's healthcare resources planning and allocation policies that are being designed for mega-cities such as Shanghai in developing countries.

## Methods

### Data collection

Considering the nearly 1-year time delay that existed in publishing Chinese official annual yearbooks, the study used secondary administrative data retrieved from the Shanghai Health Statistical Yearbook and Shanghai Health Commission and Shanghai Statistical Yearbook from 2011 to 2017 published by Shanghai Statistics Bureau. These official yearbooks reported open data annually on measures of healthcare (Shanghai Health Statistical Yearbook) and indicators of economic and social development (Shanghai Statistical Yearbook) from 16 municipal administrative divisions.

In Shanghai's 2009 medical reform, the “5 + 3+1” policy of expanding nine tertiary hospitals in the suburbs of Shanghai had set clear objectives to reinforce the utility and responsibility of public hospitals and to ameliorate the construction of the public health system. In the guidance of such policy, nine tertiary hospitals were constructed in suburban regions while a “1,560” radius of residence area accessible to healthcare services was guaranteed, so that patients in suburban and rural regions in Shanghai could easily get access to primary care institutions within 15-min walk, and to a tertiary hospital within 60-min *via* public transportation (27). Ideally, as the results showed, the healthcare resource per capita has increased in these areas, and the gap between urban and rural areas has reduced tremendously.

As a municipal city, Shanghai currently consists of 16 districts, among which are seven urban, two semi-urban, and semi-suburb, and seven suburban districts (see Supplementary Figure 1). Shanghai's urban administrative divisions are as follows: Hongkou, Huangpu, Changning, Yangpu, Xuhui, Jing'an, and Putuo. Its rural administrative divisions are Jiading, Songjiang, Chongming, Qingpu, Jinshan, Fengxian, and Baoshan. The remaining semi-urban and semi-suburb districts are Pudong New Area and Minhang. From 2010 to 2016, three administration changes have taken place in Shanghai as strategies to enhance administrative efficiency and cut administrative costs. To be more exact, in 2011, Luwan District was incorporated as the new Huangpu District; similarly, in 2015, Zhabei District was merged into Jing'an District; and Chongming County was renamed and regarded as Chongming District in 2016, suggesting an upgradation in its administrative level. To ensure the consistency of data in cross-sectional data, we reformatted the current data of the 16 administration divisions by combining the data of Luwan with that of Huangpu and the data of Zhabei with that of Jing'an in 2010.

### Measures

Five indicators of two aspects were computed and used to quantitatively measure the healthcare resource within each district of Shanghai. The facility resource was measured by the number of institutions and beds per 1,000 population. The workforce resource was measured by the number of technicians per 1,000 population, the number of doctors per 1,000 population, and the number of nurses per 1,000 population. The detailed definitions of the five indicators and the way they were calculated are demonstrated in the Supplementary material.

### Data analysis

To measure the inequality of healthcare resource allocation, many types of statistical methods are used, such as the Gini coefficient, the Theil index, and the Atkinson index, and a spatial autocorrelation analysis (28). Every method has its own strengths, for example, the Theil index can be used to measure the overall degree of differences, but it uniquely shows the contributions within a subgroup and between subgroup components on the basis of the calculated contribution rate (19, 29). In this study, both the Theil L measure and the Theil T index were calculated to measure the inequality since these indexes are the most desirable decomposition measures of inequalities and could identify different sources of inequalities. The Theil L measure is decomposable in a better sense than the Theil T, but the Theil T index is complementary to the Theil L index when there is zero population in a unit. Certainly, as the most well-recognized measure (30, 31), the Gini coefficient was also applied to calculate, with 0 representing perfect equality and 1 representing total inequality. The range of 0–0.3 means comparatively fair, and a value of 0.4 is considered to be a warning limit, while a value >0.4 indicates the existence of inequality. By comparison, spatial statistics help bring to light the equity of healthcare resource distribution for local units and can identify the distinctive administrative units, which tend to be the priority areas for promoting healthcare resource distribution equitably. To comprehensively assess the global inequality of healthcare resources for an entire region, as well as the local inequality of healthcare resources for its local units, we applied both the Theil index and the spatial autocorrelation analysis to investigate the inequality of five healthcare resource indicators in Shanghai from 2010 to 2016 as mentioned in the following sections.

### Theil index evaluation

The Theil index was applied to evaluate the inequity of healthcare resource allocation. The Theil index was initially developed to measure the inequality between distinct groups, known as the between-region difference (32). This formula can be defined as follows:


(1) $$\begin{array}{l} T=\overset{k}{\underset{i=1}{\sum }}w_{i}\;ln\left(\frac{w_{i}}{e_{i}}\right) \end{array}$$

Where *w*_*i*_ is the proportion of the income of group i accounting for the total income of all groups and *e*_*i*_ represents the proportion of the people in group i accounting for the overall population of all groups. In this study, we defined *w*_*i*_ as the proportion of healthcare resources in district i accounting for the resources of the whole city, and we defined *e*_*i*_ as the proportion of the population in district i accounting for the overall population of the city.

Furthermore, the Theil index can be divided into T_inter_ and T_intra_, and the calculation of T_inter_ and T_intra_ is as follows (2):


$$\begin{array}{l} T=T_{inter}+T_{intra} \end{array}$$

(2) $$\begin{array}{l} T_{inter}=\overset{m}{\underset{j=1}{\sum }}P_{j}\times \log\left(\frac{P_{j}}{Y_{j}}\right) \end{array}$$

(3) $$\begin{array}{l} T_{intra}=\overset{m}{\underset{j=1}{\sum }}P_{j}\times T_{j} \end{array}$$

Where P_j_ is the proportion of the three groups (urban, semi-urban and semi-suburb, and suburban regions) population accounting for the overall population of Shanghai; Y_j_ is the proportion of healthcare resources owned by the three groups accounting for the total number of healthcare resources; and T_j_ is the T of the three groups. The value of the Theil index ranges from 0 to 1, with 0 representing perfect equality and 1 representing completely unequal.

### Global and local spatial correlation evaluation

To assess the correlation between each indicator and its spatial location, Moran's index (Moran's I) and local Moran's index (local Moran's I) were calculated for global and local spatial autocorrelation, respectively (25, 32–35). Global Moran's I and the local Moran's I were used to evaluate the entire degree of spatial autocorrelation and estimate the local autocorrelation between a single position and its neighbors, respectively.

First, we constructed the appropriate spatial weight matrix which illustrates the location information of the geographical units to calculate Moran's indexes and conduct the spatial autocorrelation analysis of the target geographical units. Essentially, spatial weights can be constructed in two ways: either based on contiguity from polygon boundary files or based on the distance between points. Here, we chose contiguity-based spatial weights since our main interest lies in understanding spatial interdependence between adjacent administrative divisions. This study adopts a widely used strategy to construct the spatial weight, a binary contiguity matrix with the rook criterion, i.e., spatial neighboring criterion based on border sharing. For example, in the following formula (4), i and j are administrative units in Shanghai. If they are adjacent provincial units, the value of W_ij_ will be 1, and if these two units share no border, the value of W_ij_ will be 0. We have row-standardized the spatial weight matrix to control the influences from the number of bordering units.


(4) $$\begin{array}{l} w_{ij}=\{\begin{array}{c} 1\;ifif\;two\;geographical\;units\;i\;and\;j\;share\;borders\\ 0\;otherwise\; \end{array} \end{array}$$

The formula for calculating global Moran's I is defined as follows:


(5) $$\begin{array}{r} I=\frac{\text{n}}{\sum_{i=1}^{n}\sum_{j=1}^{n}w_{ij}}▪\frac{\sum_{i=1}^{n}\sum_{j=1}^{n}w_{ij}\left({\text{x}}_{i}-\bar{x}\right)\left({\text{x}}_{j}-\bar{x}\right)}{\sum_{i=1}^{n}{\left({\text{x}}_{i}-\bar{x}\right)}^{2}}\\ \text{i}\neq j \end{array}$$

Where n represents the total number of districts (n = 16); X_i_ and X_j_ are the density of institutions, beds, technicians, doctors, and nurses of the ith and jth district of interest; x is the average density of institutions, beds, technicians, doctors, and nurses of 16 districts; and W_ij_ refers to the spatial weight between location i and j. Global Moran's I ranges from −1 to 1 and resembles the Pearson correlation coefficient in interpretation. If a global Moran's I is above 0, it indicates a positive spatial autocorrelation (concentration tendency of similar values, high with high and low with low; the classification of high and low values depends on the mean value), and a higher value indicates stronger correlations. If a global Moran's I is < 0, it indicates a negative correlation (concentration tendency of dissimilar values, high with low). In particular, 0 means that all the high and low values are randomly distributed in space.

Similarly, the formula for calculating local Moran's I is defined as follows:


(6) $$\begin{array}{l} I=\frac{\text{n}\left({\text{x}}_{i}-\bar{x}\right)\sum_{j=1}^{n}w_{ij}\left({\text{x}}_{j}-\bar{x}\right)}{\sum_{i=1}^{n}{\left({\text{x}}_{i}-\bar{x}\right)}^{2}}\;\text{i}\neq j \end{array}$$

The explanations of parameters in formula (6) were the same as those in formula (5). Through computing local Moran's I value, the corresponding cluster map was generated to classify the spatial association into four quadrants categories referred to as high–high, low–low, low–high, and high–low, relative to the mean, which is the center of the graph.

The value of local Moran's I varies from −1 to 1. A positive value approaching 1 means a stronger geographical concentration of units with similar values (high–high, low–low; high values and low values are classified based on the mean value), while that approaching −1 stands for the opposite situation (low–high, high–low clustering patterns). If local Moran's I = 0, it means the random distribution of the units.

Theil index computation was performed using Stata statistical software version 15.0 (StataCorp LP, College Station, TX, USA). The software GeoDa was used for spatial analysis and clustering map generation.

## Results

### Inequality trends based on Theil indexes from 2010 to 2016

#### At municipal level

Comparing the respective results for each year, both Theil T and Thiel L indexes perform better for the technicians, doctors, nurses, and beds than institutions (see Table 1). This indicated that the inequality in the health workforce and hospital bed allocation was more severe than the inequality in the institution as a whole. From 2010 to 2016, except for the number of doctors, there were decreasing trends for the indexes of all the other indicators which indicated an overall decrease in the inequality in healthcare resources in Shanghai over 7 years. Similarly, the same time trends of inequality for healthcare resources at the municipal level were also seen in terms of the Gini coefficient (see Table 2). The data show that the Gini coefficients of those healthcare resources ranged between 0.2517 and 0.3874, indicating relatively proper equality, especially for health institutions. Notedly, regarding the decomposition of inequality, between-region inequality accounted for 60% or more of municipal inequality in the distribution of health workforce and bed resources for both Theil L and Theil T indexes from 2010 to 2016. However, it only explained 0.51–50% of overall inequality in the distribution of institutions for both Theil T and Theil L indexes in the same period. In contrast, within-region inequality accounted for more than half of municipal inequality in the distribution of institutions for both Theil T and Theil L indexes from 2010 to 2016, especially, it overwhelmingly accounted for the inequality of health institutions from 2013 (Theil T:89.99%; Theil L:87.90%), and kept increasing until 2016 (Theil T:99.49%; Theil L:99.48%) (see Table 3 and Supplementary Table 1).

**Table 1:** Trends of the Theil indices for the health resources in Shanghai at municipal level from 2010 to 2016.

**Indicator**	**2010**		**2011**		**2012**		**2013**		**2014**		**2015**		**2016**	
	**Theil T**	**Theil L**	**Theil T**	**Theil L**	**Theil T**	**Theil L**	**Theil T**	**Theil L**	**Theil T**	**Theil L**	**Theil T**	**Theil L**	**Theil T**	**Theil L**
IPK	0.0916	0.0891	0.106	0.1022	0.0966	0.094	0.0745	0.0727	0.0739	0.0721	0.0765	0.0746	0.0787	0.0764
BPK	0.1886	0.186	0.1895	0.1882	0.1846	0.183	0.186	0.1831	0.187	0.182	0.1938	0.1875	0.1845	0.175
TPK	0.238	0.2257	0.2299	0.2189	0.2269	0.2155	0.2137	0.2028	0.2205	0.2096	0.2207	0.2119	0.226	0.2143
DPK	0.2042	0.1923	0.2033	0.1915	0.2034	0.1917	0.1848	0.174	0.195	0.1849	0.1981	0.189	0.2065	0.1942
NPK	0.2513	0.2423	0.2467	0.2379	0.2418	0.2322	0.2323	0.2224	0.2362	0.2272	0.2398	0.2326	0.2462	0.2364

IPK, Number of institutions per 1,000 people; BPK, Number of beds per 1,000 people; DPK, Number of doctors per 1,000 people; TPK, Number of technicians per 1,000 people; NPK, Number of nurses per 1,000 people.

**Table 2:** Trends of the Gini coefficients for the health resources in Shanghai at municipal level from 2010 to 2016.

**Gini coefficient**	**2010**	**2011**	**2012**	**2013**	**2014**	**2015**	**2016**
IPK	0.2335	0.2517	0.2436	0.2158	0.2153	0.2185	0.2214
BPK	0.3409	0.3427	0.3385	0.3387	0.3382	0.3434	0.3330
TPK	0.3748	0.3692	0.3664	0.3567	0.3622	0.3629	0.3650
DPK	0.3463	0.3452	0.3454	0.3310	0.3410	0.3437	0.3481
NPK	0.3874	0.3839	0.3797	0.3725	0.3723	0.3791	0.3828

IPK, Number of institutions per 1,000 people; BPK, Number of beds per 1,000 people; DPK, Number of doctors per 1,000 people; TPK, Number of technicians per 1,000 people; NPK, Number of nurses per 1,000 people.

**Table 3:** The trends of Theil indices and their decomposition for the health resources in Shanghai from 2010 to 2016.

**Indicator**		**2010**		**2011**		**2012**		**2013**		**2014**		**2015**		**2016**	
		**Theil T**	**Theil L**	**Theil T**	**Theil L**	**Theil T**	**Theil L**	**Theil T**	**Theil L**	**Theil T**	**Theil L**	**Theil T**	**Theil L**	**Theil T**	**Theil L**
IPK	BR	0.0427	0.0430	0.0488	0.0491	0.0474	0.0478	0.0082	0.0088	0.0075	0.0082	0.0083	0.0092	0.0004	0.0004
	WR	0.0489	0.0461	0.0572	0.0531	0.0492	0.0462	0.0663	0.0639	0.0664	0.0639	0.0682	0.0654	0.0783	0.0760
	Total	0.0916	0.0891	0.1060	0.1022	0.0966	0.094	0.0745	0.0727	0.0739	0.0721	0.0765	0.0746	0.0787	0.0764
BPK	BR	0.1107	0.1149	0.1182	0.1235	0.1181	0.1234	0.1199	0.1255	0.1218	0.1275	0.1288	0.1352	0.0628	0.1274
	WR	0.0779	0.0711	0.0713	0.0647	0.0665	0.0596	0.0661	0.0576	0.0652	0.0545	0.0651	0.0523	0.1217	0.0476
	Total	0.1886	0.1860	0.1895	0.1882	0.1846	0.1830	0.1860	0.1831	0.1870	0.1820	0.1938	0.1875	0.1845	0.1750
TPK	BR	0.1516	0.1610	0.1496	0.1586	0.1481	0.1568	0.1360	0.1430	0.1447	0.1528	0.1491	0.1580	0.1519	0.1623
	WR	0.0864	0.0647	0.0803	0.0603	0.0788	0.0587	0.0777	0.0598	0.0758	0.0568	0.0716	0.0539	0.0741	0.0520
	Total	0.2380	0.2257	0.2299	0.2189	0.2269	0.2155	0.2137	0.2028	0.2205	0.2096	0.2207	0.2119	0.2260	0.2143
DPK	BR	0.1310	0.1374	0.1308	0.1373	0.1330	0.1397	0.1108	0.1150	0.1238	0.1292	0.1295	0.1358	0.1334	0.1408
	WR	0.0732	0.0549	0.0725	0.0542	0.0704	0.0520	0.0740	0.0590	0.0712	0.0557	0.0686	0.0532	0.0731	0.0534
	Total	0.2042	0.1923	0.2033	0.1915	0.2034	0.1917	0.1848	0.1740	0.1950	0.1849	0.1981	0.1890	0.2065	0.1942
NPK	BR	0.1671	0.1789	0.1628	0.1739	0.1598	0.1704	0.1522	0.1615	0.1606	0.1713	0.1676	0.1794	0.1715	0.1853
	WR	0.0842	0.0634	0.0839	0.0640	0.0820	0.0618	0.0801	0.0609	0.0756	0.0559	0.0722	0.0532	0.0747	0.0511
	Total	0.2513	0.2423	0.2467	0.2379	0.2418	0.2322	0.2323	0.2224	0.2362	0.2272	0.2398	0.2326	0.2462	0.2364

IPK, Number of institutions per 1,000 people; BPK, Number of beds per 1,000 people; DPK, Number of doctors per 1,000 people; TPK, Number of technicians per 1,000 people; NPK, Number of nurses per 1,000 people; BR, Between-region; WR, Within region.

### At regional level

Similarly, at the district level, declining trends were observed in the Theil indexes of healthcare resource allocation indicators in central and rural areas, while steady trends of these indexes were observed in semi-urban and semi-suburb areas (see Supplementary Figure 2). However, there was an exception for the Theil index of institutions in rural districts where it showed an upward trend from 2010 to 2016. Interestingly, when comparing the data from the same year, the Theil indexes of the institutions, doctors, technicians, nurses, and beds in the semi-urban and semi-suburb districts were all far lower than those in the central and rural areas. It indicated equality of healthcare resource allocation in the semi-urban and semi-suburbs was higher than that in the central and rural districts in Shanghai during the period.

### Inequality trends based on spatial autocorrelation analysis from 2010 to 2016

#### Global spatial autocorrelation

In Table 4, significant global spatial autocorrelations were found in all the indicators of health resources from 2010 to 2016 (all *p* < 0.05), with the exceptions for the number of institutions per 1,000 population from 2013 to 2016 (*p* > 0.05).

**Table 4:** Global spatial autocorrelation analyses of institutions, beds and workforce distribution from 2010 to 2016.

**Year**	**IPK**			**BPK**			**TPK**			**DPK**			**NPK**		
	**Moran's I**	**Z**	***P***	**Moran's I**	**Z**	***P***	**Moran's I**	**Z**	***P***	**Moran's I**	**Z**	***P***	**Moran's I**	**Z**	***P***
2010	−0.068	−2.718	0.006	0.117	2.212	0.024	0.079	3.526	0.003	0.090	3.582	0.003	0.087	3.595	0.002
2011	−0.090	−2.566	0.011	0.124	2.355	0.019	0.079	3.566	0.002	0.087	3.588	0.003	0.076	3.565	0.002
2012	−0.066	−2.756	0.006	0.132	2.265	0.022	0.082	3.587	0.002	0.090	3.605	0.003	0.076	3.565	0.002
2013	−0.215	−2.240	0.422	0.162	2.391	0.020	0.084	3.566	0.002	0.078	3.454	0.002	0.079	3.613	0.002
2014	−0.217	−0.280	0.411	0.164	2.339	0.022	0.105	3.712	0.002	0.099	3.634	0.002	0.107	3.746	0.002
2015	−0.231	−0.348	0.385	0.177	2.513	0.015	0.122	3.775	0.002	0.107	3.454	0.002	0.134	3.855	0.002
2016	−0.115	−0.344	0.385	0.169	2.570	0.013	0.120	3.753	0.002	0.101	3.648	0.002	0.134	3.828	0.002

IPK, Number of institutions per 1,000 people; BPK, Number of beds per 1,000 people; DPK, Number of doctors per 1,000 people; TPK, Number of technicians per 1,000 people; NPK, Number of nurses per 1,000 people.

**Figure 1:**
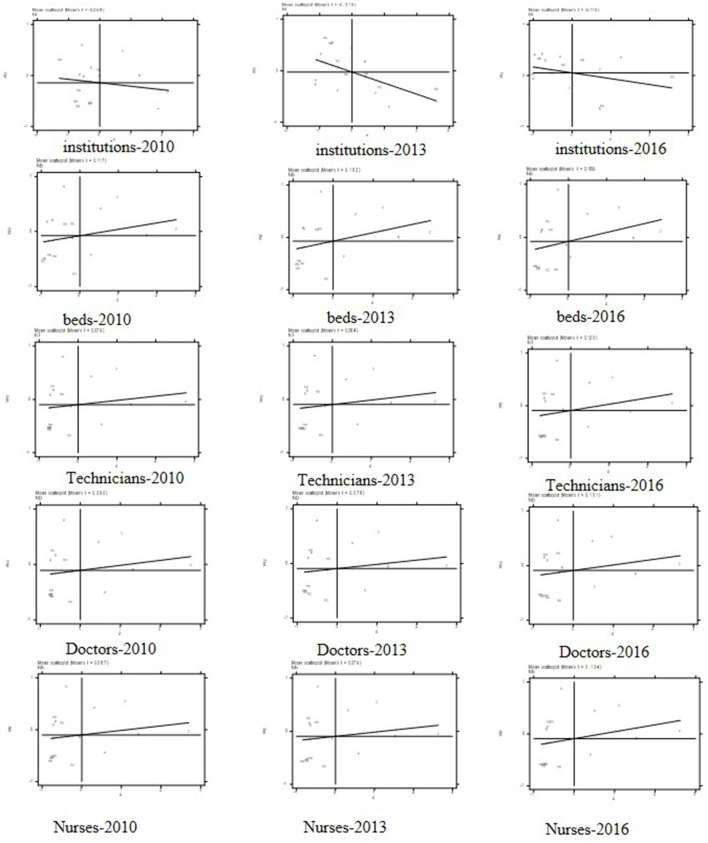
Moran's I scatterplots for institutions, beds, technicians, doctors, and nurses in 2010, 2013, and 2016.

As shown in Figure 1, most of the administrative districts were located in Quadrants II and III in 2010 for institutions, beds, and health workforce, suggesting a strongly negative and positive spatial autocorrelation, respectively. From 2010 to 2016, global Moran's I increased by certain degrees for all indicators, with a negative spatial autocorrelation in the institution and positive spatial autocorrelation in the other indicators, and it indicates an upward tendency for spatial discrepancy and autocorrelation, respectively. Notably, during the 7 years, except for institutions, the increase of the positive global Moran's I for beds, technicians, doctors, and nurses all accelerated after 2010 (Table 4). For example, global Moran's I increased by 0.049, 0.041, 0.011, and 0.047 for beds, technicians, doctors, and nurses, respectively, but negative global Moran's I decreased by 0.049 for institutions instead. In total, the increase in positive global Moran's I indicates that the relatively serious spatial autocorrelation gradually increased for beds and workforce while the decrease in negative global Moran's I indicates that spatial discrepancy increased. In addition, the number of points situated in Quadrants I and IV gradually increased for institutions, while the number of points in the same quadrants keep evidently unchanged for beds and workforce during the entire period.

### Spatial classification

**Figure 2:**
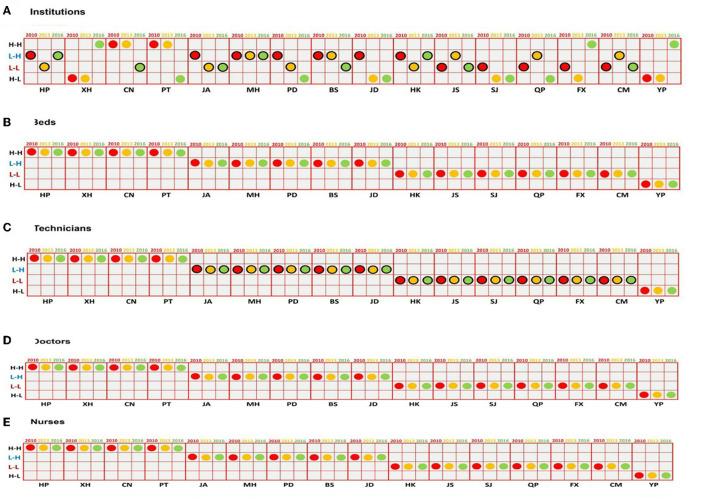
Classification of four types of spatial autocorrelation for institutions **(A)**, beds **(B)**, technicians **(C)**, doctors **(D)**, and nurses **(E)** from 2010 to 2016.

Through global spatial autocorrelation statistics, Figure 2 presents the detailed spatial classifications of 16 administrative divisions for institutions, beds, doctors, technicians, and nurses, respectively. For institutions at all the time points, most of the urban administrative districts were situated evidently in Quadrants I, II, III, and IV, whereas the rural administrative units were mainly distributed in Quadrant II (low–high type) and III (low–low type), indicating a regional disparity of the institution distribution. Notably, since 2010, the number of administrative units belonging to the low–high and low-low types decreased continually, from 7 in 2010 to 6 in 2016, and 5 in 2010 to 4 in 2016. In addition, the number of administrative units belonging to high–high and high–low groups both increased by 1 during the entire period, respectively.

Regarding beds, technicians, doctors, and nurses were all shown the same distribution, meaning still remained unchanged in the spatial autocorrelation classification, with more urban administrative districts placed in high–high type (quadrant I) and more rural administrative units located in low–high and low–low types (quadrant II and III) during the entire period. There were no number changes observed in the spatial transformation of the four types after 2009's health reform. For example, the urban administrative districts (HP, XH, CN, and PT) were evenly distributed in the four quadrants, whereas dual-semi administrative districts (MH and PD) were steadily located in Quadrant II (low–high type), and the rural administrative ones (BS, JD, JS, SJ, QP, FX, and CM) were mainly evenly distributed in two quadrants (low–high and low–low types), especially Quadrant III (low–low type) during the entire period.

### Local spatial autocorrelation

**Figure 3:**
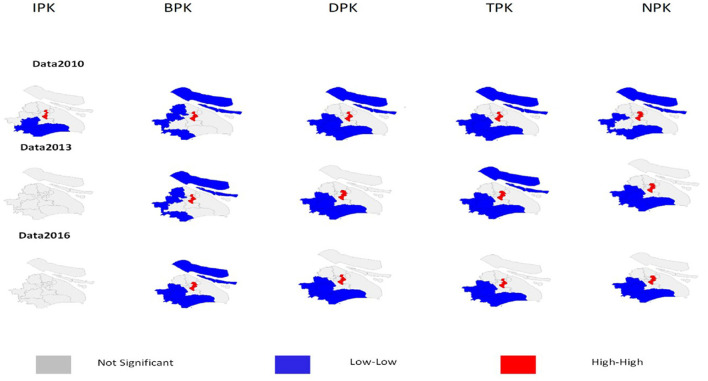
The cluster map of local Moran's I analysis from 2010 to 2016.

The univariate LISA cluster map shows that five types of healthcare resources have their own characteristics (Figure 3). For institutions in 2010, the high–high cluster units can be found in urban areas, such as JA and XH districts, while the low–low cluster units can be found in rural areas, such as SJ, JS, and FX. However, in 2013 and 2016, there was no significant clustering of “hotspot” or “coldspots” showing up in the aforementioned districts for the number of health institutions. Similarly, the high–high cluster units can be also found in urban areas for beds, such as JA, HP, and XH districts, while the low–low cluster units can be found in rural areas, such as CM, QP, JS, and FX. In addition, the complexity of high–high cluster features increased over time. HK district moved into the high–high cluster feature in 2016, while the low–low cluster feature of JD was no longer significant at the last time point. Instead, SJ had become an exhibit of a significant L–L cluster feature at the same time point.

For the health workforce, during the entire period, numbers of technicians, doctors, and nurses were shown the same cluster feature that L–L cluster areas were all located in four rural administrative districts (QP, SJ, JS, and FX), whereas the H–H cluster areas were all located in urban administrative districts, such as JA, XH, and HP. Moreover, the CM district moved out of L–L cluster areas gradually from 2010 to 2016, and HK remained unchanged in belonging to the H–H cluster type for nurses during the 7-year period. Due to space limitations, only clustering maps in 2010, 2013, and 2016 are provided in Figure 3.

### Robustness test

In order to verify the reliability of the abovementioned analyses, the robustness of results was tested using a transformation of the spatial weights matrix. Referring to the study by Parent et al. (37), the spatial adjacency matrix was replaced by the geographical inverse distance weight matrix. The results on time trends of spatial autocorrection of healthcare resources and the significance of the results were mainly consistent with the original analyses, which confirmed that the findings of the present study have satisfactory robustness. Detailed results are presented in Supplementary Table 2 and Supplementary Figures 3, 4.

## Discussion

This study explored time trends of inequality of distribution in healthcare resources at the municipal level and district level in Shanghai from 2010 to 2016. It confirmed the reduction of the inequalities of medical resources distribution in Shanghai after 2009's new reform. However, we also investigated the severe situation of inequality of doctor allocation at the municipal level and health institution allocation at the rural level and further explored spatial changing patterns and geographic clustering features that existed across 16 districts during these 7 years by using the spatial autocorrelation method.

First, inequality of healthcare resource allocation was largely improved; however, some types of healthcare resources were unevenly distributed at the municipal level (i.e., doctors) or at the rural level (i.e., institutions), and these disparities persisted, even becoming more severe during the period. The Theil indexes of indicators were improved both at the municipality level and regional level during 7 years, indicating that the Shanghai government's targeted policies, especially the rural-area-targeted “5 + 3+1” program, have greatly reduced the inequality in healthcare resource allocation. However, the inequalities in some types of healthcare resources are still preserved. Furthermore, the results showed that the main inequality is found between regions for the workforce, and within regions for institutions. It reminded us to rethink the limited policy impacts. Though the targeted policies were given to the rural areas to reduce the inequality in healthcare resource allocation, the problems of maldistribution of physicians had been unsolved due to the long-term training cycle for a health workforce and other social factors that contribute to the workforce maldistribution. For health institutions in rural districts, their lack of competitive salary levels and socioeconomic conditions constrained them to attract and maintain a high-quality health workforce. Hence, though the expansion and opening of branch institutions of tertiary hospitals in the urban districts of Shanghai can be easily observed and achieved in rural districts, workforce shortage has limited the volume and quality of services. Severely, the inequality in institution density in rural areas was worsened by blindly expanding the health facilities, since the policy preferences to build institutions were often given to the priority rural areas that more easily developed compared to the other underdeveloped rural areas. This resulted in an unfair distribution of institutions among rural areas. Some similar studies were in line with these findings (2, 36). Therefore, region-level policy depending on the types of healthcare resources may be most suitable to address these inequity issues, and more detailed area-specific healthcare resource planning and allocation guidelines are in urgent need to reallocate healthcare resources. For example, on the one hand, the government should promote the hierarchical diagnosis and treatment strategy, improve the wages and other SES for the health workforce in primary health institutions, especially in suburban districts, and encourage urban regions with relatively abundant health workforce to transfer healthcare human resources into suburban areas with insufficient health human resources to meet the unmet needs. On the other hand, the government should also formulate criteria for healthcare resource allocation, construct health institutions, and conduct health investments reasonably depending on every district's economic development and population health needs.

Second, we further used the spatial autocorrelation analysis method to reveal the spatial changing patterns of healthcare resource distribution across different districts and identified priority areas in Shanghai from 2010 to 2016. To the best of our knowledge, the Theil index method could measure the gaps among all the units, while spatial autocorrelation analysis is used to identify how the spatial distribution of these gaps. In this study, using the latter method, we illustrated different spatial concentration statuses of five types of healthcare resource distribution across Shanghai from 2010 to 2016. Through global spatial autocorrelation analysis, it was found that the strengthening of spatial concentration effect in healthcare resources of beds and workforce in Shanghai over time was obvious. This distribution pattern was also proved by the results with the local spatial autocorrelation analysis that the H–H type clusters were all located in traditional central administrative districts and L–L type clusters were all located in rural ones for these four types of healthcare resources during the 7-year period. They all implied that economic strength and geographic location have a positive effect in determining healthcare resource distribution. Compared to other regions, economically strong central urban districts experience shortages of beds and healthcare workers and are better equipped to deal with these shortages of healthcare resources. To solve the problem of maldistribution of healthcare resources, especially in the health workforce, Shanghai's health reform had helped disadvantaged districts to improve their health resource shortage from 2010 to 2016. For example, the “coldspots” districts of institution density including Songjiang, Jinshan, and Fengxian (displayed as low–low cluster areas in Figure 3) were no longer “coldspots” in 2016, indicating an improvement in the distribution of institutions. Similarly, such low–low clustering of bed density in the Jiading district, workforce density in the Chongming district disappeared gradually from 2010 to 2016. However, some urban areas showing high–high clustering and rural areas showing low–low clustering of different kinds of health resource density co-existed from the beginning to the end of the study. This indicated that healthcare resource concentration tended to be correlated and agglomerated together. The aforementioned findings further added evidence of the existence of geographic imbalances in healthcare resource distribution in Shanghai. Under the background of regional economic integration, theoretically, the concentration of medical resources in different provinces and cities should be spatially correlated (2, 38). As a mega-city, Shanghai's urban areas are no exception and more attractive to the health workforce due to their comparative economic, cultural, educational, and professional advantages. The suburban areas are less economically developed, and their longer geographic distance to health facilities and poorer public transportation will result in lower access to healthcare services for patients there, and fewer career opportunities for health professionals in their institutions, which in turn further made them more difficult in displacing from the low–low cluster area. To solve this problem, in addition to suburban areas' efforts to mitigate on their own, such as multi-sectoral alliances from related governmental sectors, mutual assistance between undeveloped regions is also needed. For instance, the government could implement continuous and sustainable multisectoral and intersectoral policies and initiatives to overcome conflicting interests between sectors, power imbalances, and competition for medical resources. These initiatives would require the integration among health sectors, public health sectors, and other social service agencies such as the public transportation sector, toward the aim of improving access to care for residents in suburban areas. In addition, regional alliance medical centers in undeveloped regions could be set up to expand the capacity of high-quality medical resources and promote the allocation balance of healthcare resources between urban and rural areas.

Third, this study also sharpened the focus of illustrating the most in need of healthcare resources in the most in need of regional units. We further detected the priority areas that should be first considered to make efforts to distribute rational healthcare resources to improve the equity of healthcare resources in Shanghai. In this study, for beds and workforce, the high–high cluster units can be found in JA, HP, and XH districts, and the low–low cluster units can be found in the southwest and south part of Shanghai, especially QP, SJ, JS, and FX (Figure 3). It also indicated that disparity of beds and workforce still kept unchanged in these areas after the 2009's health reform. These findings of the study by spatial clustering provide more solid evidence for area-specific and subtype-specific workforce policy-making in Shanghai, aiming at the rural beds and health workforce, by prioritizing the identified low–low and low–high cluster areas. For example, some point-to-point cross-regional medical pairing-assistance programs should be suggested to allocate healthcare resources between JA, HP, and XH districts, which have shortages of beds and workforce, and QP, SJ, JS, and FX districts, which were abundant in these healthcare resources. Such pairing programs will make it more accurate and efficient by paring up the geographical regions of greatest need and the relative abundance of certain subtypes of the health workforce. In addition, some remote medical technology can also be applied to fill the workforce allocation gaps between urban and rural districts in Shanghai (39).

The present study has some caveats that should be acknowledged. First, we measured healthcare resource allocation with five quantitate indicators without considering their quality aspects. Therefore, quality indicators measuring healthcare resource distribution should be developed in future to complement this study. Second, for data availability, the study used municipal-level and district-level data from the officially published yearbooks to measure the inequality of healthcare resources from 2010 to 2016, which may cause the problems of the modifiable areal unit problem (MAUP) (40). Data at the county level or community level would be ideal to sketch the geographic distribution of healthcare resources in future studies. Moreover, a future study on changes in healthcare resource allocation, along with comparisons with the present study, can be carried out when the data from 2017 to the present are available.

## Conclusion

This study demonstrated the trends of inequality in health institutions, beds, technicians, doctors, and nurses in Shanghai from 2010 to 2016. It further confirmed the effectiveness of Shanghai's government's new health reform since 2009 to reduce the inequality of their allocation at the municipal level. However, the inequalities of healthcare resource allocation including both between-region inequity and within-region inequality across Shanghai's 16 districts were still preserved after the new health reform. Furthermore, this study shed light on priority areas for institutions, beds, and workforce allocation through the spatial-temporal transformation of healthcare resource distribution. Since geographical factors and economic strength play important roles in the inequality in health resource distribution, to further balance the distribution of institutions, beds, and workforce and improve the equality of its allocation, it requires not only joint efforts from disadvantaged spatial adjacent units, especially L–L type cluster and L–H type cluster units to make healthcare resource allocating and planning policies from health sectors (e.g., cross-regional medical paring-assistance programs and remote medical technology) but also full consideration across all the policies and regional cooperation from economic, and human resource sectors to alleviate the factors that cause the inequality in health resource allocation in metropolises such as Shanghai in China.

## Data availability statement

The raw data supporting the conclusions of this article will be made available by the authors, without undue reservation.

## Author contributions

ED, XS, SZ, LZ, TW, and WG designed the study, acquired the data, developed the statistical plan, and drafted and revised the manuscript. TX and TW carried out the survey. ED and XS performed the statistical analysis. All authors have read and approved the final manuscript.
