# Supplementary figures and images for: Measuring the inequalities in healthcare resource in facility and workforce: A longitudinal study in China
Source: Front Public Health. 2023 Mar 16;11:1074417. doi: 10.3389/fpubh.2023.1074417 (PMC10060654; doi:10.3389/fpubh.2023.1074417)

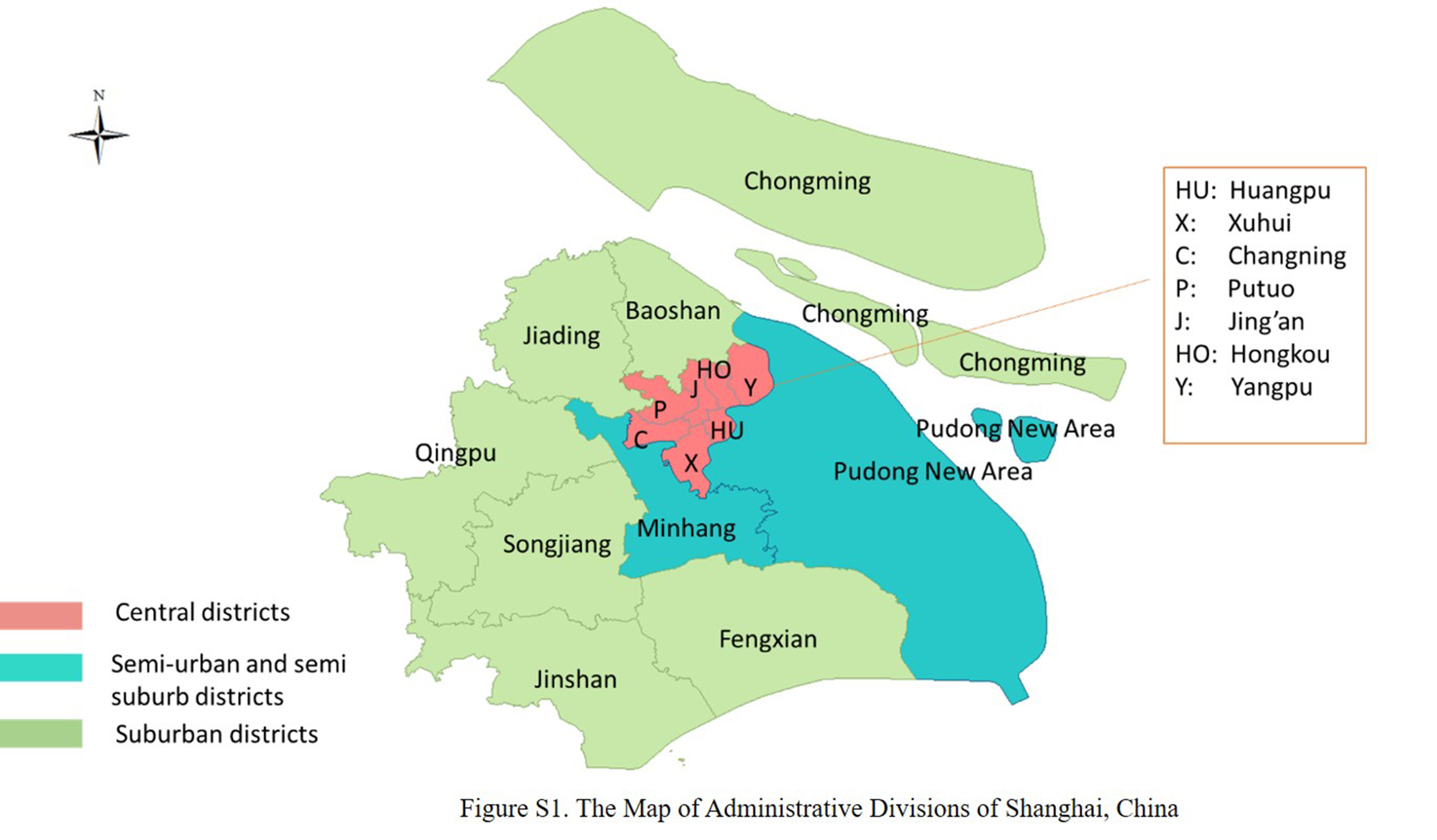

Supplement: Supplementary file 1 [file Image_1.JPEG]

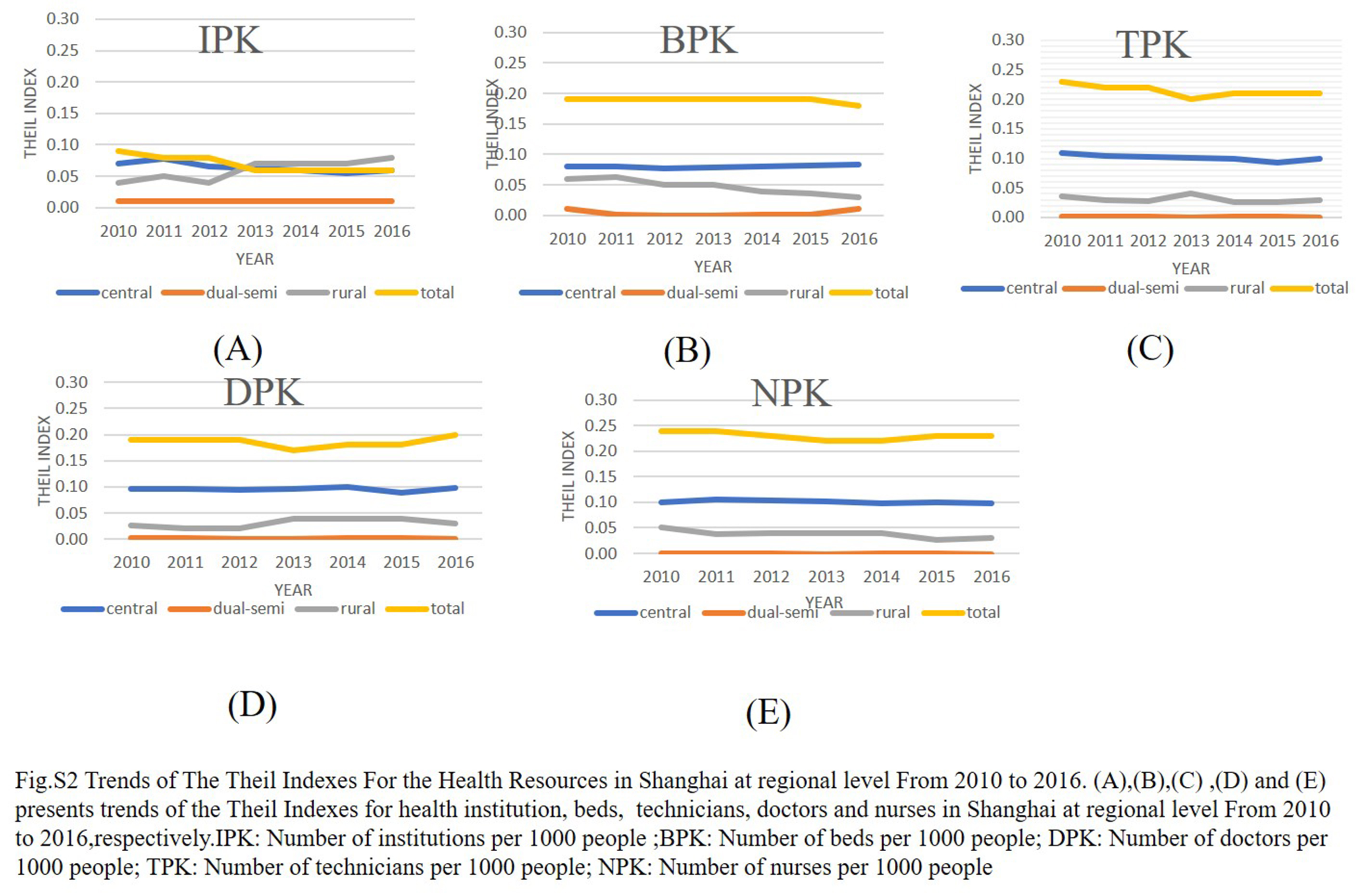

Supplement: Supplementary file 2 [file Image_2.JPEG]

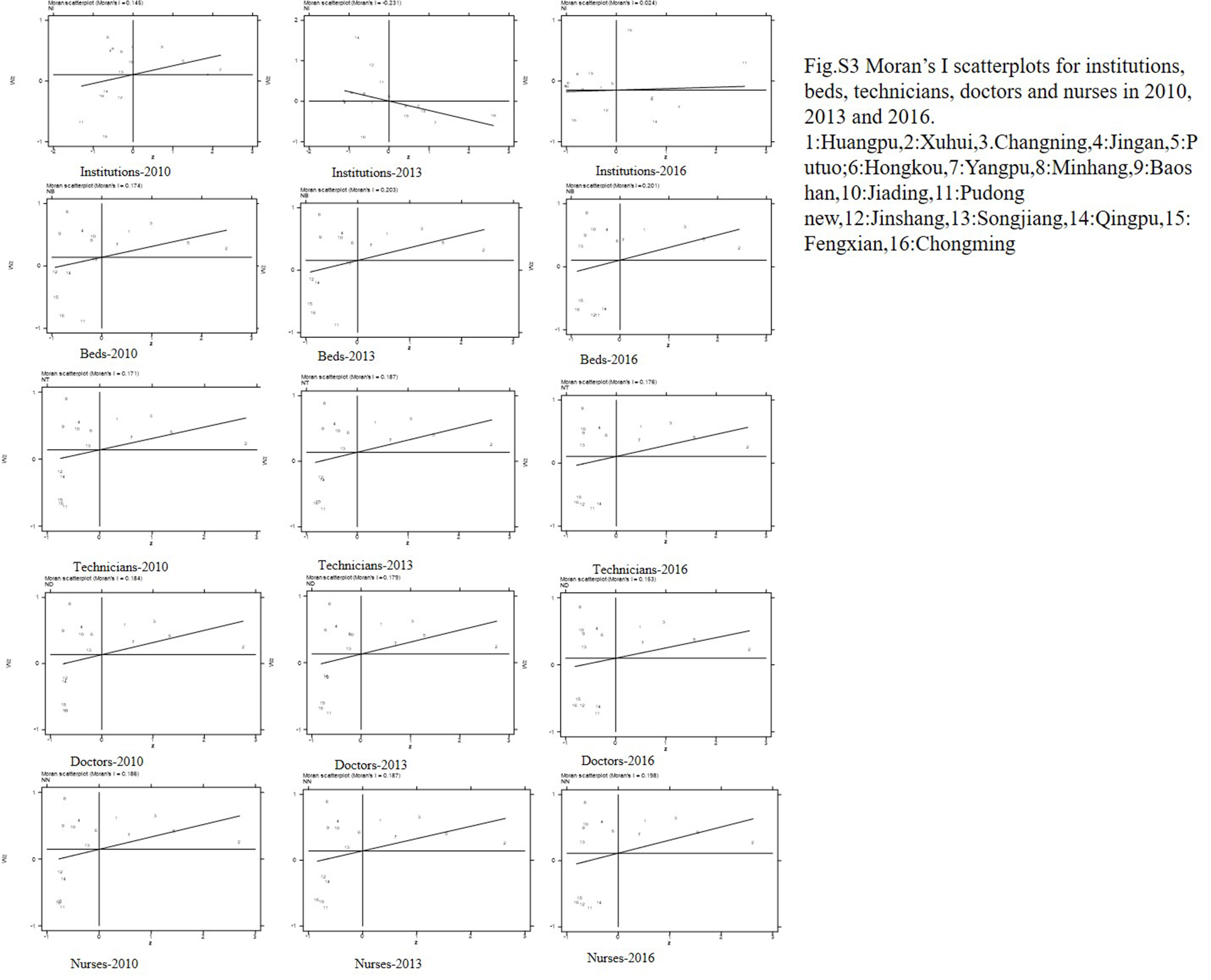

Supplement: Supplementary file 3 [file Image_3.JPEG]

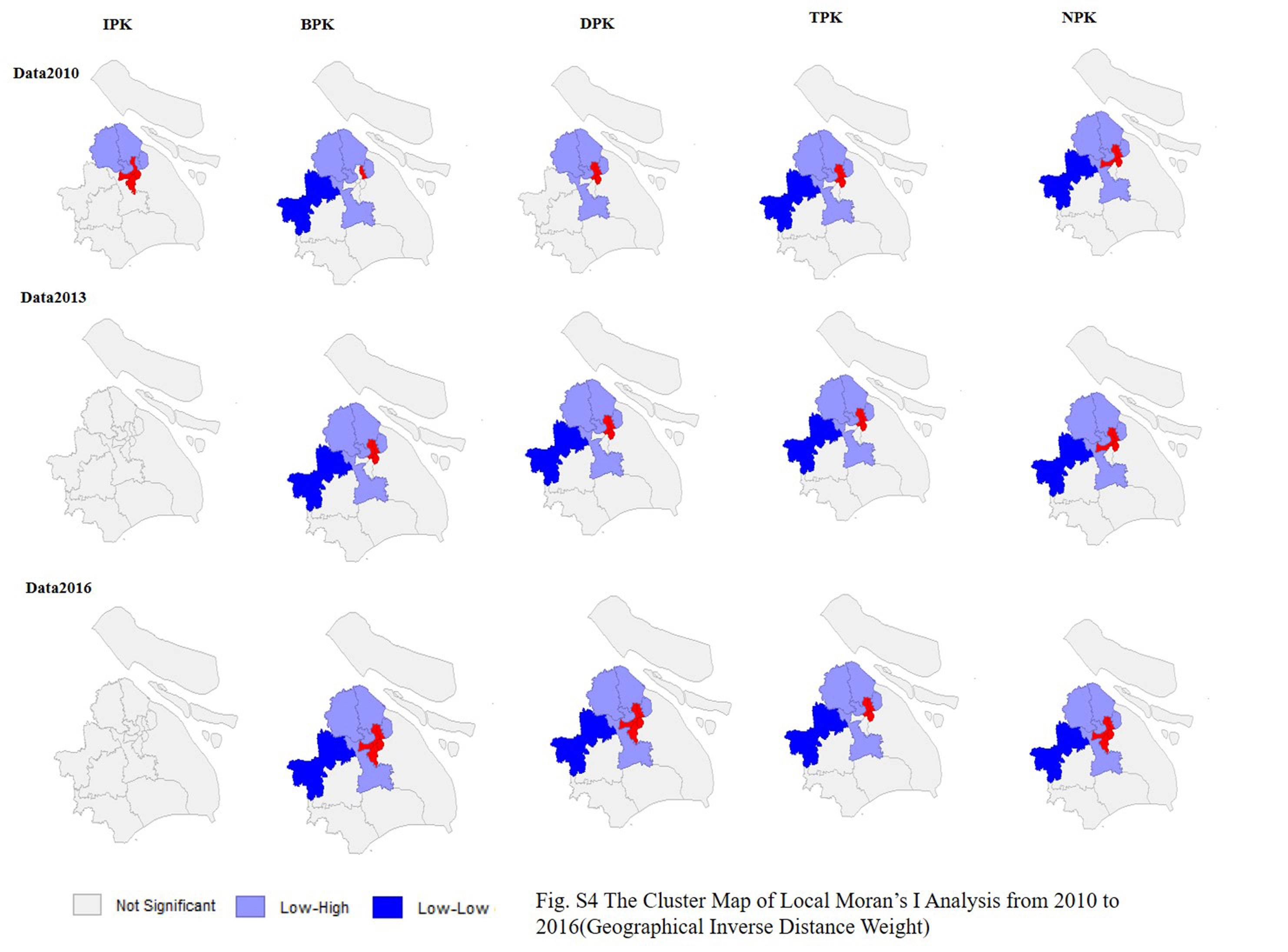

Supplement: Supplementary file 4 [file Image_4.JPEG]
