# Supplementary material for: Measuring the inequalities in healthcare resource in facility and workforce: A longitudinal study in China
Source: Front Public Health. 2023 Mar 16;11:1074417. doi: 10.3389/fpubh.2023.1074417 (PMC10060654; doi:10.3389/fpubh.2023.1074417)
Supplement: Supplementary file 5 [file Table_1.DOCX]

**Table S1 Decomposition of overall inequity by region for the healthcare resources in Shanghai From 2010 to 2016**

| Indicator | Inequality Contribution | 2010 | | 2011 | | | 2012 | | | 2013 | | | 2014 | | | 2015 | | | 2016 | | |
| --- | --- | --- | --- | --- | --- | --- | --- | --- | --- | --- | --- | --- | --- | --- | --- | --- | --- | --- | --- | --- | --- |
|  |  | Theil T | Theil L | | Theil T | Theil L | | Theil T | Theil L | | Theil T | Theil L | | Theil T | Theil L | | Theil T | Theil L | | Theil T | Theil L |
| IPK | BR | 46.62% | 48.26% | | 46.04% | 48.04% | | 49.07% | 50.85% | | 11.01% | 12.10% | | 10.15% | 11.37% | | 10.85% | 12.33% | | 0.51% | 0.52% |
|  | WR | 53.38% | 51.74% | | 53.96% | 51.96% | | 50.93% | 49.15% | | 88.99% | 87.90% | | 89.85% | 88.63% | | 89.15% | 87.67% | | 99.49% | 99.48% |
|  | Total | 100.00% | 100.00% | | 100.00% | 100.00% | | 100.00% | 100.00% | | 100.00% | 100.00% | | 100.00% | 100.00% | | 100.00% | 100.00% | | 100.00% | 100.00% |
| BPK | BR | 58.70% | 61.77% | | 62.37% | 65.62% | | 63.98% | 67.43% | | 64.46% | 68.54% | | 65.13% | 70.05% | | 66.46% | 72.11% | | 65.96% | 72.80% |
|  | WR | 41.30% | 38.23% | | 37.63% | 34.38% | | 36.02% | 32.57% | | 35.54% | 31.46% | | 34.87% | 29.95% | | 33.59% | 27.89% | | 0.00% | 27.20% |
|  | Total | 100.00% | 100.00% | | 100.00% | 100.00% | | 100.00% | 100.00% | | 100.00% | 100.00% | | 100.00% | 100.00% | | 100.00% | 100.00% | | 100.00% | 100.00% |
| TPK | BR | 63.70% | 71.33% | | 65.07% | 72.45% | | 65.27% | 72.76% | | 63.64% | 70.51% | | 65.62% | 72.90% | | 67.56% | 74.56% | | 67.21% | 75.73% |
|  | WR | 36.30% | 28.67% | | 34.93% | 27.55% | | 34.73% | 27.24% | | 36.36% | 29.49% | | 34.38% | 27.10% | | 32.44% | 25.44% | | 32.79% | 24.27% |
|  | Total | 100.00% | 100.00% | | 100.00% | 100.00% | | 100.00% | 100.00% | | 100.00% | 100.00% | | 100.00% | 100.00% | | 100.00% | 100.00% | | 100.00% | 100.00% |
| DPK | BR | 64.15% | 71.45% | | 64.34% | 71.70% | | 65.39% | 72.87% | | 59.96% | 66.09% | | 63.49% | 69.88% | | 65.37% | 71.85% | | 64.60% | 72.50% |
|  | WR | 35.85% | 28.55% | | 35.66% | 28.30% | | 34.61% | 27.13% | | 40.04% | 33.91% | | 36.51% | 30.12% | | 34.63% | 28.15% | | 35.40% | 27.50% |
|  | Total | 100.00% | 100.00% | | 100.00% | 100.00% | | 100.00% | 100.00% | | 100.00% | 100.00% | | 100.00% | 100.00% | | 100.00% | 100.00% | | 100.00% | 100.00% |
| NPK | BR | 66.49% | 73.83% | | 65.99% | 73.10% | | 66.09% | 73.39% | | 65.52% | 72.62% | | 67.99% | 75.40% | | 69.89% | 77.13% | | 69.66% | 78.38% |
|  | WR | 33.51% | 26.17% | | 34.01% | 26.90% | | 33.91% | 26.61% | | 34.48% | 27.38% | | 32.01% | 24.60% | | 30.11% | 22.87% | | 30.34% | 21.62% |
|  | Total | 100.00% | 100.00% | | 100.00% | 100.00% | | 100.00% | 100.00% | | 100.00% | 100.00% | | 100.00% | 100.00% | | 100.00% | 100.00% | | 100.00% | 100.00% |

IPK: Number of institutions per 1000 people ;BPK: Number of beds per 1000 people; DPK: Number of doctors per 1000 people; TPK: Number of technicians per 1000 people; NPK: Number of nurses per 1000 people;BR: Between-region;WR:Within region.

**Table S2 Global spatial autocorrelation analyses of institutions, beds and workforce distribution from 2010 to 2016 based on geographic inverse distanceWeight**

| Year | IPK | | | BPK | | | TPK | | | | DPK | | | | NPK | | |
| --- | --- | --- | --- | --- | --- | --- | --- | --- | --- | --- | --- | --- | --- | --- | --- | --- | --- |
|  | Moran's I | Z | P | Moran's I | Z | P | Moran's I | Z | P | Moran's I | | Z | P | Moran's I | | Z | P |
| 2010 | 0.145 | -3.354 | 0.007 | 0.174 | 2.581 | 0.014 | 0.171 | 5.642 | 0.001 | 0.184 | | 3.719 | 0.002 | 0.186 | | 3.709 | 0.001 |
| 2011 | 0.130 | -3.259 | 0.008 | 0.188 | 2.662 | 0.026 | 0.181 | 3.674 | 0.004 | 0.185 | | 3.708 | 0.002 | 0183 | | 3.775 | 0.004 |
| 2012 | 0.159 | -3.725 | 0.004 | 0.192 | 2.672 | 0.025 | 0.185 | 3.690 | 0.001 | 0.190 | | 3.733 | 0.003 | 0.186 | | 3.694 | 0.002 |
| 2013 | -0.231 | -1.088 | 0.277 | 0.203 | 2.752 | 0.020 | 0.187 | 3.700 | 0.009 | 0.179 | | 3.682 | 0.003 | 0.187 | | 3.500 | 0.004 |
| 2014 | -0.224 | -1.030 | 0.303 | 0.205 | 2.752 | 0.020 | 0.200 | 3.781 | 0.005 | 0.202 | | 3.804 | 0.001 | 0.206 | | 3.809 | 0.001 |
| 2015 | -0.220 | -1.008 | 0.313 | 0.210 | 2.794 | 0.023 | 0.211 | 3.840 | 0.002 | 0.206 | | 3.711 | 0.003 | 0.219 | | 3.890 | 0.002 |
| 2016 | 0.024 | -0.595 | 0.552 | 0.201 | 2.745 | 0.031 | 0.176 | 3.623 | 0.003 | 0.153 | | 3.482 | 0.004 | 0.198 | | 3.669 | 0.002 |

IPK: Number of institutions per 1000 people ;BPK: Number of beds per 1000 people; DPK: Number of doctors per 1000 people; TPK: Number of technicians per 1000 people; NPK: Number of nurses per 1000 people.
